# Acetabular cartilage abnormalities in elderly patients with femoral neck fractures

**DOI:** 10.1051/sicotj/2022022

**Published:** 2022-06-14

**Authors:** Hironori Ochi, Hideo Kobayashi, Tomonori Baba, Riko Nakajima, Yasuha Kurita, Suguru Kato, Kyoko Sasaki, Masahiko Nozawa, Sung-Gon Kim, Yuko Sakamoto, Yasuhiro Homma, Kazuo Kaneko, Muneaki Ishijima

**Affiliations:** 1 Department of Orthopaedic Surgery, Juntendo University Nerima Hospital 3-1-10, Takanodai Nerima-ku Tokyo 177-8521 Japan; 2 Department of Orthopaedic Surgery, San-ikukai Hospital 3-20-2 Taihei Sumida-ku Tokyo 130-0012 Japan; 3 Department of Orthopaedic Surgery, Juntendo University School of Medicine 2-1-1 Hongo Bunkyo-ku Tokyo 113-0033 Japan

**Keywords:** Femoral neck fracture, Acetabulum, Cartilage, Abnormalities, Hemiarthroplasty, Total hip arthroplasty

## Abstract

*Introduction*: Both hemiarthroplasty (HA) and total hip arthroplasty (THA) are widely accepted surgical procedures for hip replacement following displaced femoral neck fractures. However, in cases involving an intact joint line before surgery, the choice between HA and THA remains debatable. This study investigated the prevalence of acetabular cartilage and labral abnormalities in elderly patients with femoral neck fractures. *Methods*: Thirty-seven patients underwent hip arthroplasty for femoral neck fractures between April 2020 and February 2021. After excluding 4 patients, 33 patients (6 men and 27 women; mean age = 82.2 [range = 67–98] years) with fractures in 12 left and 21 right hips were included. After femoral head removal during arthroplasty, the acetabulum was macroscopically examined for the presence of cartilage and labral lesions. Acetabular cartilage abnormalities were classified as either overall degeneration or partial damage according to the cartilage damage classification system. *Results*: Acetabular cartilage abnormalities, including overall degeneration or partial damage, were found in all hips (100%). Out of the 33 hips, overall degeneration, partial damage, and labral abnormalities were detected in 32 (96.9%), 16 (48.4%), and 9 (27.2%) hips, respectively. *Discussion*: In this study, most elderly patients with femoral neck fractures exhibited acetabular cartilage and labral abnormalities, which were already present at the time of surgery. Therefore, surgeons should carefully examine these abnormalities as they may impact postoperative outcomes such as pain and function.

## Introduction

The size of the elderly population has been increasing as the global population ages, resulting in an increased incidence of osteoporotic hip fractures [1]. Any treatment for femoral neck fractures aims to return the patient to a satisfactory functional status as quickly as possible with minimal morbidity and mortality and to minimize the need for reoperation [2]. Arthroplasty is now routinely performed in older patients because of the risk of avascular necrosis of the femoral head [3]. Both hemiarthroplasty (HA) and total hip arthroplasty (THA) are widely accepted surgical procedures for hip replacement following displaced femoral neck fractures [1]. However, in cases involving an intact joint line before surgery, the choice between HA and THA remains debatable [3]. Several investigations based on radiographic images have shown that intracapsular fractures rarely occur in arthritic hips [4, 5]. However, although no gross anatomical or radiographic abnormalities suggestive of advanced osteoarthritis are present, labral damage and cartilage lesions have been frequently observed in the anterosuperior periphery of the acetabulum in elderly patients who had undergone HA for displaced femoral neck fractures [6].

After 4 years of follow-up, a higher acetabular erosion risk has been observed following HA rather than THA; it is often associated with persistent pain and the need for reoperation [1, 2, 7]. Some previous studies reported no significant differences in postoperative acetabular erosion rates between HA and THA within 1 year of follow-up, and similar results were observed after 2 years of follow-up [1, 7]. Nevertheless, several studies reported that HA, as a treatment modality for femoral neck fractures, is inferior to THA in terms of hip pain and function during the first postoperative year and thereafter [7–9]. These results raise questions on whether there were acetabular cartilage or labral abnormalities before surgery. The articular cartilage undergoes molecular structural changes during aging, and some changes resemble those seen in osteoarthritis-related degeneration [10]. Thus, we hypothesized that cartilage abnormalities exist in the periphery of the acetabulum and the overall acetabulum of elderly patients with femoral neck fractures. In this study, we aimed to investigate the prevalence of such abnormalities in the acetabular cartilage and labrum of elderly patients with femoral neck fractures.

## Patients and methods

### Patients

Thirty-seven patients underwent hip arthroplasty for femoral neck fractures at two orthopedic institutions between April 2020 and February 2021. The inclusion criteria were age ≥60 years and osteoporotic femoral neck fractures resulting from low-energy trauma. The exclusion criteria were as follows: (1) age <60 years; (2) history of hip surgery; (3) advanced hip osteoarthritis (grade ≥2 according to the Kellgren–Lawrence classification system [11]); (4) acetabular dysplasia (lateral center-edge angle [LCE angle] of ≤25°); (5) inflammatory diseases, such as rheumatoid arthritis; (6) history of femoral neck fractures for >4 weeks; and (7) pathological fractures. Four patients were excluded for the following reasons: age <60 years at the time of surgery, presence of rheumatoid arthritis, having a >4-week history of femoral neck fractures and having a pathological fracture. Finally, as shown in Table 1, 33 patients (6 men and 27 women; mean age = 82.2 [range = 67–98] years) with fractures in 12 left and 21 right hips were included. All patients underwent HA or THA according to the surgeon’s preoperative decision based on patient age, pre-injury walking ability, and general condition. Patient’s age, body mass index (BMI), sex, fracture side, and Garden classification based on the radiographic assessment of the degree of displacement of femoral neck fractures [12], LCE, and Sharp angles as radiographic acetabular coverage measurements [13, 14], surgical approach, and arthroplasty type are presented in Table 1.

**Table 1:** Patient characteristics.

	*n* = 33
Mean age, years (*SD*)	82.2 (8.0)
Mean body mass index, kg/m^2^ (*SD*)	20.1 (3.3)
Sex, *n* (%)	
Male	6 (18.1%)
Female	27 (81.8%)
Fracture side, *n* (%)	
Left	12 (36.3%)
Right	21 (63.6%)
Garden classification, *n* (%)	
Type I	0 (0%)
Type II	3 (9.0%)
Type III	10 (30.3%)
Type IV	20 (60.6%)
Mean LCE angle, degrees (*SD*)	31.3 (4.7)
Mean Sharp angle, degrees (*SD*)	38.8 (3.2)
Surgical approach, *n* (%)	
Posterior approach	2 (6.0%)
Direct lateral approach	2 (6.0%)
Anterolateral approach in the supine position	4 (12.1%)
Direct anterior approach	25 (75.7%)
Type of arthroplasty, *n* (%)	
Hemiarthroplasty	8 (24.2%)
Total hip arthroplasty	25 (75.7%)

*SD*: standard deviation; LCE angle: lateral center-edge angle.

### Evaluation

**Figure 1:**
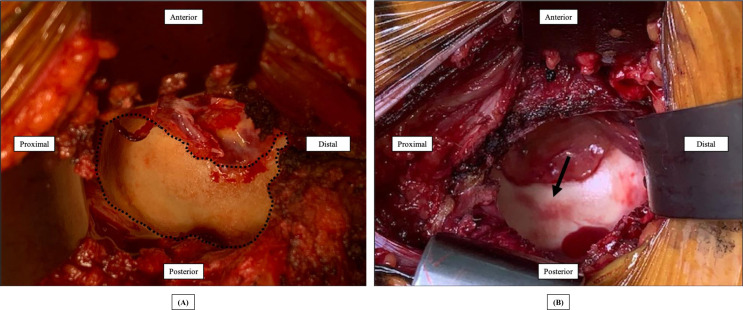
(A) Overall degeneration in the acetabular cartilage (demarcated by the dotted line). (B) Partial damage in the acetabular cartilage (black arrow). Both macroscopic views of the acetabulum show the right hip via the direct anterior approach.

After femoral head removal during arthroplasty, the acetabulum was macroscopically examined for cartilage and labral lesions. Two senior orthopedic surgeons (HO or HK), who were experienced in hip joint replacement, performed the evaluations. All acetabula were documented photographically. Acetabular cartilage abnormalities were classified as either overall degeneration or partial damage based on the cartilage damage classification developed by Beck et al. [15]. Overall degeneration was defined as the presence of surface roughening, fibrillation, or thinning in the overall acetabular cartilage (Figure 1A), whereas partial damage was defined as the presence of partial thinning, full-thickness defects, or deep fissuring in the bone in an area of the acetabular cartilage (Figure 1B). Labral abnormalities included separation from the bony acetabular rim, tears, hypertrophy, and scarring [6]. If the labrum could not be evaluated because it could not be observed clearly, the status was defined as unknown. The presence and location of partial acetabular cartilage damage and labral lesions were documented using the geographic zone method [16]. All findings were converted to correspond to the right side.

### Intra- and inter-observer reliability

The intra- and inter-observer reliability in evaluating acetabular cartilage abnormalities was assessed using intraoperative photography. Twenty-four cases with clear photographic images were randomly selected for the intra- and inter-observer reliability assessment. Two orthopedic surgeons (HO, involved in treatment and intraoperative evaluation, and YK, not involved in these steps) conducted the intra- and inter-observer reliability tests. They evaluated the acetabular cartilage photographs, and the accuracy rates between the intraoperative and photographic findings were calculated as the intra- and inter-observer reliability, respectively. The calculations were performed using the kappa coefficient as follows: no agreement ≤ 0; poor agreement = 0.01–0.20; fair agreement = 0.21–0.40; moderate agreement = 0.41–0.60; substantial agreement = 0.61–0.80; and almost perfect agreement = 0.81–1.00 [17]. The kappa values corresponding to the intra- and inter-observer reliability in evaluating acetabular cartilage abnormalities were 0.91 and 0.83, respectively.

To evaluate the labral abnormalities, it was necessary to touch and assess the labrum via intraoperative probing; therefore, intra- and inter-observer reliability tests could not be performed.

### Statistical analyses

Statistical analyses were performed using STATA statistical software (version 14.2; Stata Inc., College Station, TX, USA). Continuous variables were expressed as mean ± standard deviation, whereas categorical variables were expressed as numbers and ratios. Partial acetabular cartilage damage and labral abnormalities were assigned numbers and ratios (the number of lesions divided by the total number of lesions) correlating with their zone. If multiple lesions were found within one zone in a patient, the number of lesions was defined as one lesion per zone. Independent-sample *t*-test and Fisher’s exact test were used to compare the differences in clinical and radiographic features (age, BMI, sex, fracture side, Garden classification, LCE angle, and Sharp angle) between patients with posterior partial damage lesions located in zone 4 or 5 (posterior partial damage group) and those without these lesions (no posterior partial damage group). Results were considered statistically significant at *p* < 0.05.

## Results

**Figure 2:**
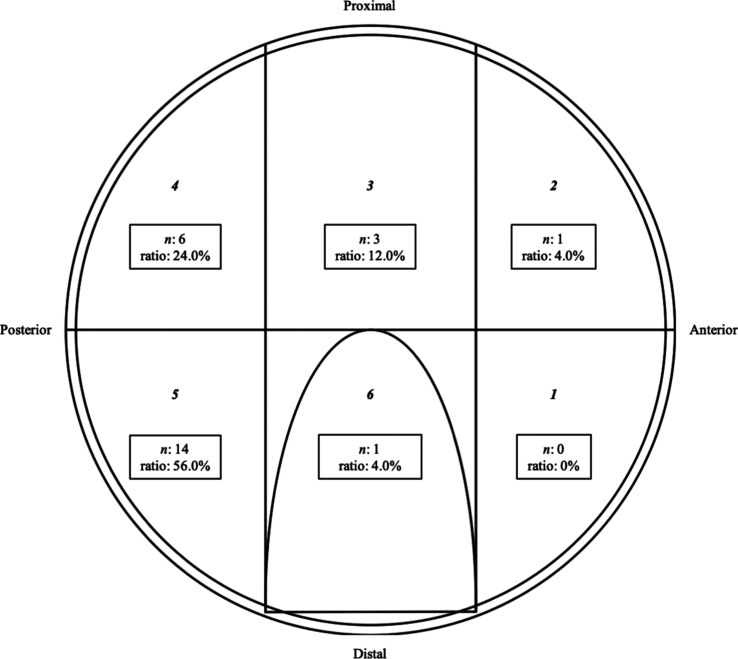
The presence and location of partial damage in the acetabular cartilage documented using a geographic zone method. The partial damage lesions in the acetabular cartilage were assigned numbers and ratios (the number of lesions divided by the total number of lesions) correlating with their zone. Overall, 25 lesions of partial damage were found in 16 hips.

The classification of acetabular cartilage abnormalities is presented in Table 2. Acetabular cartilage abnormalities, including overall degeneration or partial damage, were found in all hips (100%); overall degeneration was detected in 32 (96.9%) out of 33 hips, whereas partial damage was identified in 16 (48.4%) hips exhibiting 25 partial damage lesions. The locations of partial damage are presented in Figure 2. The majority of cases with partial damage had lesions in the posterior-inferior zone of the acetabulum. Furthermore, posterior partial damage lesions located in zone 4 or 5 were found in 15 (45.4%) out of the 33 hips examined (posterior partial damage group) (Table 3). The mean Sharp angle was significantly larger in the posterior partial damage group than in the no posterior partial damage group (*p* = 0.021) (Table 3). Apart from the Sharp angle, no significant differences in clinical and radiographic features were observed between these groups (Table 3).

**Table 2:** Prevalence of abnormalities in the acetabular cartilage and labrum.

	*n* = 33
Abnormalities in the acetabular cartilage, *n* (%)	
Overall degeneration or partial damage	33 (100%)
Overall degeneration	32 (96.9%)
Partial damage	16 (48.4%)
Labral abnormalities, *n* (%)	
Abnormalities	9 (27.2%)
None	12 (36.3%)
Unknown	12 (36.3%)

**Table 3:** Comparison of clinical and radiographic features between the posterior partial damage and no posterior partial damage groups.

	Posterior partial damage group (*n* = 15)	No posterior partial damage group (*n* = 18)	*p* value
Mean age, years (*SD*)	82.6 (8.5)	81.8 (7.8)	0.772^a^
Mean body mass index, kg/m^2^ (*SD*)	20.8 (2.9)	19.4 (3.6)	0.288^a^
Sex, *n* (%)			
Male	2 (13.3%)	4 (22.2%)	0.665^b^
Female	13 (86.6%)	14 (77.7%)	
Fracture side, *n* (%)			
Left	6 (40.0%)	6 (33.3%)	0.731^b^
Right	9 (60.0%)	12 (66.6%)	
Garden classification, *n* (%)			
Type I	0 (0%)	0 (0%)	0.431^b^
Type II	1 (6.6%)	2 (11.1%)	
Type III	3 (20.0%)	7 (38.8%)	
Type IV	11 (73.3%)	9 (50.0%)	
Mean LCE angle, degrees (*SD*)	30.0 (4.1)	32.3 (5.1)	0.165^a^
Mean Sharp angle, degrees (*SD*)	40.2 (3.1)	37.6 (2.9)	0.021^a^

a Independent-sample *t*-test;

b Fisher’s exact test.

*SD*: standard deviation; LCE angle: lateral center-edge angle.

**Figure 3:**
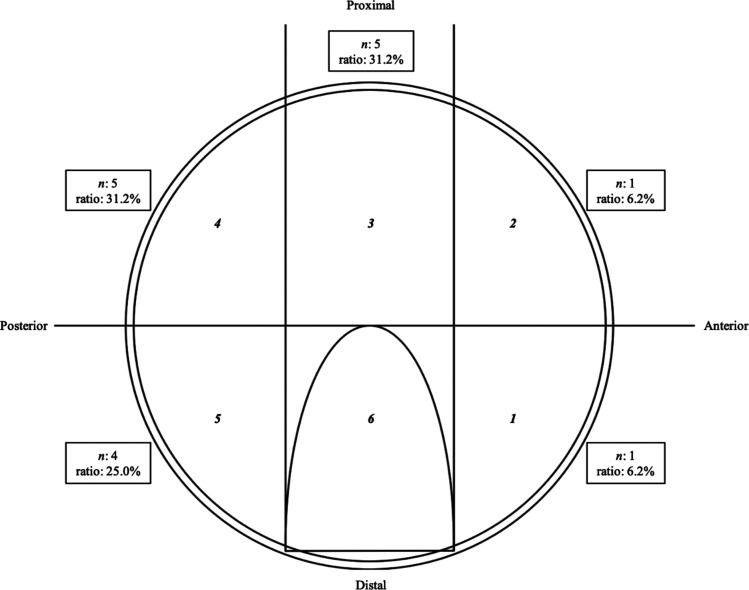
The presence and location of labral lesions documented using a geographic zone method. The labral abnormality lesions were assigned numbers and ratios (the number of lesions divided by the total number of lesions) correlating with their zone. Overall, 16 labral abnormalities were found in 9 hips.

Labral abnormalities were found in 9 (27.2%) of the 33 hips, with a total of 16 labral abnormalities (Table 2). The locations of labral abnormalities are shown in Figure 3. Most were observed in the middle-superior and posterior-superior acetabular zones; however, the cases classified as unknown were excluded from the count.

## Discussion

All elderly patients with femoral neck fractures included in this study exhibited acetabular cartilage abnormalities, and labral abnormalities were also confirmed in some cases. These factors may influence postoperative outcomes, such as pain and function after arthroplasty. Therefore, surgeons should consider these results before choosing between HA and THA to treat femoral neck fractures in elderly patients.

Overall acetabular cartilage degeneration was found in 96.9% of the patients included in this study, possibly indicating degeneration due to aging. Osteoarthritis, an age-related disease, is the most common joint disorder and the leading cause of chronic disability, highly prevalent in the elderly [18]. Wei et al. [19] used high-field magnetic resonance imaging (MRI) T2 mapping to examine the femoral head cartilage changes in healthy young and elderly patients with femoral neck fractures and elderly patients with osteoarthritis. This technique was selected owing to its potential for investigating early degenerative cartilage changes [19, 20]. The researchers found higher signal intensities and T2 values in the cartilage of elderly patients with femoral neck fractures than those in the cartilage of healthy young patients; on the other hand, they observed lower signal intensities and T2 values in the cartilage of elderly patients with femoral neck fractures than those in the cartilage of elderly patients with osteoarthritis [19]. Furthermore, they demonstrated that the cellular and molecular hip cartilage changes observed in elderly patients with femoral neck fractures largely resembled those seen in early osteoarthritis [19]. Although high-field MRI T2 mapping and in vitro analyses of the acetabular cartilage were not performed in the current study, a macroscopic evaluation of acetabular cartilage degeneration could be performed intraoperatively by referring to Beck et al.’s cartilage damage classification [15].

Partial acetabular cartilage damage was found in 48.4% of the patients in this study, with confirmed labral abnormalities in some cases. Moreover, the mean Sharp angle was significantly larger in the posterior partial damage group than in the no posterior partial damage group. The potential injury mechanism causing partial acetabular cartilage damage and labral abnormalities are impingement between the femoral head and neck and acetabular rim. Leunig et al. [6] reported that acetabular rim degeneration is common in aged hips and appears to be triggered by femoroacetabular impingement. Yang et al. [21] reported that anterior femoroacetabular impingement is associated with femoral neck fractures. Both reports noted that femoroacetabular impingement could result in acetabular rim damage localized in the anterosuperior quadrant of the acetabulum [6, 21]. However, compared with the findings of other reports [6, 21], in this study, partial acetabular cartilage damage and labral abnormalities were more frequently observed in the posterior region of the acetabulum. Rivière et al. [22] reported that the loss of lumbar lordosis and pelvic retroversion resulting from spine aging caused increased functional acetabular anteversion. While this prevents the risk of anterior impingement, it causes hip-spine syndrome involving posterior impingement [22]. This study reported radiographically-measured larger Sharp angles and smaller LCE angles compared to those reported in previous studies [6, 21], which potentially indicates pelvic retroversion in a high proportion of cases [13]; this posterior impingement mechanism might have been the underlying cause in a greater percentage of cases in this study than was found in previous studies [6, 21]. Furthermore, Henebry and Gaskill [13] reported that pelvic retroversion increases radiographic measurements of the Sharp angle, which was less affected by pelvic tilt compared to other radiographic acetabular coverage measurements. On the other hand, since the LCE angle is largely affected by the pelvic tilt [13], the large variation in the values obtained in this study may suggest that there was no significant difference in the LCE angle between the posterior partial damage group and the no posterior partial damage group. Therefore, a larger Sharp angle may be considered a useful preoperative index for assessing the presence of partial acetabular cartilage damage in the posterior region of the acetabulum.

We acknowledge several limitations of our study. First, because the direct anterior approach (DAA) and anterolateral approach in the supine position were used more often than other approaches, it was difficult to intraoperatively evaluate the anterior part of the acetabular rim. If this part could have been observed intraoperatively, the number of hips with partial acetabular cartilage damage or labral abnormalities might have increased. Additionally, the ratios of these locations would likely change. Furthermore, when the labrum could not be reliably observed, it was marked as unknown. Despite these limitations, our hypothesis remains valid. Second, THA was performed more frequently than HA based on the surgeons’ preoperative decisions. Some previous studies reported that THA was a preferable treatment option to HA in healthy elderly patients with displaced femoral neck fractures owing to better functional outcomes and lower reoperation rates [1, 9]. However, dislocation is a major concern after primary THA for intracapsular femoral neck fractures [1, 2], some previous studies reported on the effectiveness of THA via DAA for femoral neck fractures with a low risk of dislocation [23, 24]. These factors influenced the surgeons’ preference for THA in the current study. Consequently, there might have been a measurement bias during the acetabular cartilage assessment. However, the intra- and inter-observer reliability test results suggest that the influence of measurement bias was small. Third, hip morphological features, such as the femoral head-neck offset, cam deformity, and alpha angle, were not evaluated because macroscopic and radiographic evaluations of hip joint anatomical morphology on the fractured side were difficult, if not impossible, to perform. Investigating the pre-injury hip joint morphological abnormalities would have yielded interesting results. Finally, although determining how acetabular cartilage and labral abnormalities influence postoperative clinical outcomes would have been insightful, postoperative clinical outcomes were not evaluated. Tracking clinical outcomes appears to be more important in HA than in THA; therefore, further investigations are warranted.

## Conclusion

In this study, most elderly patients with femoral neck fractures exhibited acetabular cartilage and labral abnormalities, which were already present at the time of surgery. When arthroplasty is chosen as the treatment for femoral neck fractures, surgeons should take these findings into consideration when deciding between HA and THA.
